# Adherence and Sociodemographic Determinants of Adherence to the Mediterranean Diet among Slovenian Adults and the Elderly

**DOI:** 10.3390/nu15143219

**Published:** 2023-07-20

**Authors:** Tamara Poklar Vatovec, Zala Jenko Pražnikar, Ana Petelin

**Affiliations:** Faculty of Health Sciences, University of Primorska, Polje 42, SI-6310 Izola, Slovenia; tamara.vatovec@fvz.upr.si (T.P.V.); zala.praznikar@fvz.upr.si (Z.J.P.)

**Keywords:** Mediterranean diet, adherence, adults, elderly, sociodemographic parameters, food groups, health

## Abstract

The Mediterranean diet (MD) is considered a model for good health, and is promoted worldwide as one of the healthiest dietary patterns. Despite the MD’s health benefits, the literature suggests that adherence to the MD tends to be in decline in most populations worldwide, including those in the Mediterranean region. The aim of this study was to investigate adherence to the MD, and its main sociodemographic and lifestyle factors, in the Slovenian population. Using a nationwide cross-sectional food consumption survey (SI.Menu), data were collected from a general questionnaire, from the 14-item MD adherence screener (14-MEDAS score), and from a questionnaire on the dietary habits of 850 adults and elderly people. The mean MEDAS score for the total study sample was 5.6 (SD 2.1), indicating a low adherence to the MD among the Slovenian population. The adherence to the MD was higher among women (OR = 1.534; 95% Cl 1.156–2.034), those with a university degree (OR = 1.527; 1.098–2.125; compared to those with no university degree), those who lived in a suburb or city (OR = 1.511; 1.016–2.249; OR = 1.568; 1.122–2.191; compared with those who lived in a village), non-smokers (OR = 1.561; 1.380–1.830; compared with smokers), and those who lived in the western part of Slovenia (OR = 1.558; 1.170–2.074; compared with those who lived in eastern Slovenia). Adherence to the MD in the Slovenian population is low, and is strongly related to educational level, gender, geographic region, place of residence, and smoking status. The frequency of the consumption of different food groups is also closely related.

## 1. Introduction

The Mediterranean diet (MD) is considered a model of good health, and is promoted worldwide as one of the healthiest diets. The reason is that it is said to be a diet extremely rich in monounsaturated fatty acids, fibre, antioxidants, vitamins, and minerals [1]. The MD represents the traditional Cretan diet before 1960, and differs from the modern diet mainly in the use of foods that are locally grown, seasonal, fresh, and minimally processed. In 2010, it was recognized as an Intangible Cultural Heritage of Humanity by UNESCO. The word comes from the Greek word “diaita” which means “way of life”, and refers to a set of skills, knowledge, rituals, symbols, and traditions that extend from the landscape to the table. The MD is defined by the consumption of fruit and vegetables, nuts, olives and olive oil (as the main source of fat), legumes, and fish, a moderate consumption of wine with meals, and smaller amounts of red meat, cured meat products, refined dairy products, and full-fat dairy products. In addition, salt is partially replaced by the use of spices and herbs [1,2]. In 2010, the Food and Agriculture Organization of the United Nations (FAO) included it in its charter, and recognized it as one of the best sustainable forms of nutrition for the planet. The FAO and the World Health Organization (WHO) define sustainable, healthy diets, including the MD, as dietary patterns that promote all dimensions of individual health, have a low environmental impact, and are accessible and affordable, safe and fair, and culturally acceptable [3].

The MD has been associated with beneficial effects on human health since the beginning. Indeed, many studies have shown that a greater adherence to the MD reduces the risk of cardiovascular diseases, neurodegenerative diseases, diabetes, and all-cause mortality, and reduces mortality from cancer [4,5,6,7,8,9]. In the last 25 years, an impressive number of different questionnaires to assess adherence to the MD have been proposed [10]. The PREDİMED consortium established a 14-point Mediterranean Diet Adherence Screener (MEDAS) questionnaire [11]. This tool includes 12 questions on the frequency of food consumption, and 2 questions on the typical eating habits of Hispanic people. The authors found that the MEDAS questionnaire was a valid tool for the rapid prediction of adherence to the MD, and that it might be practical in clinical use [12].

However, despite the health benefits of the MD, the literature suggests that adherence to the MD tends to be in decline in most populations worldwide, including those in the Mediterranean region [13,14,15,16]. Industrialization, urbanization, the globalization of the food market, and economic development are changing our eating habits [17]. Nowadays, a lack of time, poor dietary habits, and food of a poorer nutritional quality, together with a sedentary lifestyle lead to an increased risk of obesity and other non-communicable diseases [18]. In Slovenia, which is located in Central Europe, touches the Alps, and borders the Mediterranean Sea, the rates of overweight and obesity are higher than in most EU countries [19].

Therefore, the main objective of this study was to estimate the actual adherence to the MD in a large sample of the adult and elderly Slovenian population, recruited from the entire Slovenian territory within the Slovenian Nutrition Survey (SI.MENU), and to investigate the main demographic, socioeconomic, and health-related factors and dietary habits that may be associated with adherence to the MD.

## 2. Materials and Methods

### 2.1. Study Design and Subjects

The study was conducted in the period from March 2017 to April 2018, as part of a nation-wide cross-sectional food consumption survey named SI.Menu 2017/18. The methodology of the survey followed the EFSA guidance on European Union Menu Methodology [20]. The details about the methodology and sample characteristics are presented elsewhere [21]. Briefly, the sample size consisted of 1,520 selected individuals with their residence in Slovenia, and the sample was selected using the Central Register of Population of Slovenia. The sample was stratified according to sex, and the age groups of adults (from 18 to 64 years old) and elderly (from 65 to 74 years old). Moreover, the age groups were also stratified, according to the size and type of settlement (6 classes), and according to the statistical region (12 regions). Institutionalized individuals (hospitalized or living in different types of institution), individuals residing abroad, individuals who experienced disease during the survey period, and those with a severe known physical or mental disability, were excluded from the study. Survey interviewers visited the selected individuals. The interviewer met each participant at least once, to provide information on the complete dietary survey and the supporting questionnaires, and to perform the anthropometric measurements. Data were collected via a face-to-face questionnaire, using computer-assisted personal interviewing (CAPI). The survey was completed by a total of 850 participants (385 adults and 450 elderly had fully completed the questionnaire data). The National Medical Ethics Committee of the Republic of Slovenia (KME 0120-337/2016) approved the study protocol. Before the start of the first home visits, all participants were informed about the study, and their written informed consent was obtained.

### 2.2. Questionnaires and Measures

Two questionnaires were prepared for the SI.Menu survey, a general questionnaire and a food propensity questionnaire. The general questionnaire, food propensity questionnaire, and anthropometric measurements were collected at the first face-to-face interview. Participants completed a general questionnaire, consisting of the following questions. (1) Health status (Did you have, or have you, any of the following diseases? Available answers were: (a) No, (b) Yes—in the last 12 months, (c) Yes—more than 12 months ago, (d) I don’t know, (e) I don’t want to answer). (2) Smoking status (current smoker, former smoker, non-smoker). (3) Socio-demographic and socio-economic determinants, such as (a) academic level: (i) none, (ii) primary school, (iii) secondary school, (iv) university, (v) masters or Ph.D.; (b) employment status: (i) employed, (ii) unemployed, (iii) student, (iv) retired; (c) questions about age and gender; (d) marital status: (i) single (single, widowed, divorced) and (ii) married or in a relationship; (e) region (west Slovenia, East Slovenia); (f) rural/urban area; (g) household size; (h) questions related to the self-estimated socio-economic standard of the household and the amount of monthly income of the respondent’s household. (4) Physical activity: (a) Are you physically active on average for at least 30 min/day or a total of 150 min/week (yes/no). The answers were directly entered into a portable computer.

Moreover, the food propensity questionnaire was used to collect the usual frequency of consumption of specific foods in the last 12 months. A list of 75 food items, some rarely consumed and some common in the Slovenian diet, corresponding to nine food groups, was used (cereals and cereal products; milk and milk products; fruit; vegetables; meat, fish, eggs, and meat products; fats and fatty food; sugar and sweeteners; beverages; miscellaneous). The frequency response options for the food list were never, 1–3 times per month or less, once per week, 2–3 times per week, 4–6 times per week, and 1–2 times per day or more.

Each participant’s body mass and height were measured at their home, in light clothing and without shoes. The body mass was measured to the nearest 0.1 kg, using a calibrated digital mass scale with a bioimpedance analyser (Tanita BC-730, Tokyo, Japan). The height was measured to the nearest 0.5 cm, using a measuring tape and wooden corner block, with the head positioned in the Frankfort horizontal plane. Moreover, the body fat, body water level, muscle mass, and bone mass were measured to assess the body composition, using a calibrated digital mass scale with a bioimpedance analyser (Tanita BC-730, Tokyo Japan), using the included algorithms. Using the weight and height data, the subject’s body mass index (BMI) was computed. The cut-off points for overweight and obesity were set at 25 kg/m^2^ and 30 kg/m^2^, respectively.

### 2.3. Adherence to the Mediterranean Diet

The level of adherence to the MD was evaluated using the 14-item Mediterranean Diet Adherence Screener (14-MEDAS) used in the PREDIMED study [22]. The MEDAS questionnaire included 14 questions, to evaluate the amount of 12 main components consumed, and two food habits related to the MD. For each item, an affirmative response was scored as one point, while a negative response corresponded to zero points (Table 1). Thus, the score of the MEDAS questionnaire ranged from 0 to 14 points. For the categorization of the adherence to the MD, we applied the following criteria: low (0–5), and moderate to high (6–14) [11].

### 2.4. Statistical Analysis

Data are presented as a frequency (%) (categorical variables) or as mean ± SD (continuous variables). Before statistical analysis, the data were checked for normally distribution. The normality of the data distribution was rejected through the Kolmogorov–Smirnov test. To explore the differences between genders, the parametric independent *t*-test, non-parametric Mann–Whitney test, or chi-squared test was used. Moreover, the linear regression analysis was used to test the influence of the sociodemographic characteristics of participants, and the frequency of consuming different food groups, on the MEDAS score, where the MEDAS score was used as a dependent variable, while different sociodemographic parameters and the most representative foods from different food groups were used as the independent variables. Moreover, to test the influence of the MEDAS score on the anthropometric parameters and health conditions of a subject, the MEDAS score was used as the independent variable, while the risk factors (anthropometric parameters and health risk factors) were used as the dependent variables. Furthermore, all values that showed significant associations with the MEDAS score were included in univariate and multivariate logistic regression analyses, to quantify the predictive ability for the adherence to the MD. For the latter, the participants were grouped according to “low” or “medium to high” adherence to the MD. The adherence to the MD (low/moderate to high) was used as a dependent variable, and the socio-demographic determinants or selected food from different food groups were used as independent variables alone (univariate) or together (multivariate). The results of the logistic regression analyses were presented as an odds ratio (OR), and their corresponding 95% confidence interval (Cl). A *p*-value less than 0.05 was considered statistically significant. All statistical analyses were performed using IBM SPSS version 23 (SPSS Inc., Chicago, IL, USA).

## 3. Results

### 3.1. Characteristics of Participants and Adherence to the Mediterranean Diet

The characteristics of the study sample, divided by gender, are shown in Table 2. A total of 850 subjects completed the entire questionnaires. The mean age of the subjects was 57.0 ± 15.8 years. The gender ratio of men to women was 52.2% to 47.8%, and the ratio of adults to elderly was 46.2% to 53.8%. The mean BMI was 27.8 ± 5.3 kg/m^2^, where 61.2% of females and 72.4% of males were overweight or obese. Most women and men lived in rural areas (49.5% and 60.6%, respectively), and the remaining participants lived in urban (approximately 30%) and suburban areas (approximately 20%). In terms of location, 60.6% of the subjects were from eastern Slovenia; the rest (39.4%) were from the western part of Slovenia. The majority of women (49.3%) and men (62.6%) had completed secondary school, 26.4% of women and 14.8% of men had completed elementary school, and 24.4% of women and 22.4% of men had a university degree or higher. The largest proportion of women (59%) and men (61.5%) were retired, 27.5% of women and 29.1% of men were employed, 8.8% of women and 6.2% of men were unemployed, and a minority of women (4.7%) and men (3.2%) were students. Half of the subjects reported a low family income. When women and men were compared, smoking habits were found to differ, with more than two-thirds of the women being non-smokers. Statistically significant differences between women and men were found in some anthropometric measurements, with the body weight significantly higher in men (*p* < 0.001), and the percentage of body fat statistically higher in women (*p* < 0.001). As for general health, most subjects were apparently healthy. As shown in Table 2, hypertension and hypercholesterolemia were the most frequently reported chronic diseases. Statistically significant differences between women and men were found for hypertension, type 1 diabetes mellitus, cardiovascular disease, and osteoporosis.

Statistically significant differences were also found between women and men in the MEDAS scores and adherence to the MD. Women had significantly higher scores, and a higher adherence to the MD, compared with men. The mean MEDAS score for the total study sample was 5.6 (SD 2.1), indicating a low adherence to the MD in the Slovenian population.

### 3.2. The Frequency of Consumption of the Different Food Groups

The frequency of consumption of the different food groups is shown in Table 3. Taking into account only the gender, women consume milk (*p* = 0.007), yoghurt (*p* < 0.001), soured milk and cottage cheese (*p* < 0.001), fruit (*p* < 0.001), unsalted nuts (*p* = 0.008), vegetables (*p* = 0.010), fish (*p* = 0.044), olive oil (*p* = 0.004), and butter (*p* = 0.002) more frequently than men. On the other hand, women are less likely to consume white bread (*p* = 0.018), pasta (*p* = 0.031), cheese (*p* = 0.005), beef and veal (*p* < 0.001), sausages and hot dogs (*p* < 0.001), hamburgers (*p* < 0.001), wine (*p* < 0.001), and convenience foods (*p* = 0.024), compared to men. In fact, almost 51% of women consume milk 1–2 times per day or more (men 40%), almost 68% consume fruit 1–2 times per day or more (men 50%), almost 66% consume vegetables 1–2 times per day or more (men 45%), and almost 40% consume olive oil 1–2 times per day or more (men 27%). On the other hand, more than one-third of men consume white bread 1–2 times per day or more (one-fourth of women), nearly 15% consume beef 4–6 times or more per week (women only 5%), nearly 60% consume sausages and hot dogs 1–3 times or more per week (women only 40%), 35% consume hamburgers 1–3 times or more per week (women only 2%), and nearly 45% consume prepared foods 1–3 times or more per week (women only 29%). In addition, as expected, men still drink more wine than women; 10% of men drink wine 1–2 times per day or more (women about 3%), 8% of men drink wine 4–6 times per week (women about 3%), and 33% of men drink wine once or 2–3 times per week (women 22.5%).

In general, we Slovenians are partially following the guidelines of eating red meat no more than two to three times per week (76.6% of participants eat red meat up to three times per week) and white meat (poultry meat) up to three times per week (77.4% of participants consume poultry up to three times per week). In contrast, we are far from meeting the recommendation to eat fish. In our study population, only 22.9% of participants met the guidelines of eating fish at least once or twice a week. On average, we consume too much meat and, in particular, too many meat products (53% of men eat sausages and hot dogs 2–3 times/week) and convenience foods (hamburgers (7.6% of men 2–3 times/week) and pizza (17.0% of men 2–3 times/week)), while our fish consumption is too low. In addition, we are far from meeting the recommendation to eat at least five servings of fresh fruit and vegetables per day. In our study group, only 50% of participants (55% of women and 45% of men) consumed vegetables, and only 60% of participants (67% of women and 50.5% of men) consumed fruit daily. The consumption of olive oil is also very low. Only 32.8% of participants (38.8% of women and 26.8% of men) consumed olive oil daily.

### 3.3. The Influence of the Sociodemographic Characteristics of Participants and Frequency of Consumption of Different Food Groups on the MEDAS Score

A linear regression analysis was used to test the influence of the sociodemographic characteristics of participants on the MEDAS score. The regression model explained only 7.9% of total variance, and was statistically significant (F = 7.619; *p* < 0.001). The regression coefficients are presented in Table 4. The statistically significant coefficients are marked in bold. Associations (*p* < 0.05) were found for gender, physical activity, place of residence, education, geographical area, and smoking status, supporting the extent to which these variables were used to create the MEDAS score. The variables with the highest positive regression coefficients were as follows: (1) education, (2) gender, (3) geographical area, and (4) physical activity. The variable with negative regression coefficients was the smoking status.

Moreover, the frequency of consumption of different food groups explained 60.8% of total variance, and was statistically significant (F = 53.045; *p* < 0.001). The regression coefficients are presented in Table 5. Associations (*p* < 0.05) were found for white bread, fresh fruit, unsalted nuts, fresh vegetables, beans and peas, poultry, beef and veal, sausages and hot dogs, fish, olive oil, butter, margarine, wine, and prepared food, supporting the extent to which these variables were used to create the MEDAS score. The variables with the highest positive regression coefficients were as follows: (1) olive oil, (2) fresh fruit, (3) beans and peas, (4) fresh vegetables, (5) wine, (6) poultry, (7) unsalted nuts, and (8) fish. The variables with the highest negative regression coefficients were as follows: (1) butter, (2) beef and veal, (3) sausages and hot dogs, (4) margarine, (5) white bread, and (6) prepared food.

### 3.4. Association between Adherence to the Mediteranean Diet and Amthropometric and Socio-Demographic Variables

A univariate and multivariate logistic regression was used to quantify the predictive ability for the adherence to the MD. The univariate and multivariate logistic regression revealed that women were more likely to be moderately to strongly MD-compliant, compared with men. Being female was associated with 68.9% odds in the univariate (*p* < 0.001) and 48.2% odds in the multivariate of higher adherence to the MD (*p* = 0.008). In addition, the univariate and multivariate logistic regression revealed that participants living in the city and in a suburb (with 72.1% odds for a suburban area, and with 71.4% for the city in univariate analysis (*p* = 0.005), and with 51.1% odds for a suburban area, and with 56.8.4% for the city in the multivariate analysis (*p* < 0.001)), those who had a university degree (with 70.3% odds in the univariate analysis (*p* = 0.001), and with 58.8% odds in the multivariate analysis (*p* = 0.010)), those who lived in the western part of Slovenia (with 69.0% odds in the univariate analysis (*p* < 0.001), and with 44.7% odds in the multivariate analysis (*p* = 0.014)), and who were not smokers are more likely to be moderately to highly MD-adherent, compared to participants who lived in a village, or in the eastern part of Slovenia, did not have a university degree, and were smokers (Table 6).

In addition, the univariate logistic regression for the frequency of consumption of different food groups revealed that participants who ate white bread (*p* < 0.001), beef and veal (*p* = 0.018), sausages and hot dogs (*p* < 0.001), hamburgers (*p* = 0.031), margarine (*p* < 0.001), and prepared foods (*p* = 0.005) less frequently were more likely to be moderately to severely MD-adherent. The multivariate logistic regression analysis also revealed that participants who ate beef and veal (*p* = 0.018), sausages and hot dogs (*p* < 0.001), and butter (*p* = 0.005) less frequently were moderately to strongly MD-adherent. In addition, the univariate logistic regression analysis revealed that participants who were more likely to eat brown bread (*p* < 0.001), yoghurt, soured milk and cottage cheese (*p* < 0.001), cheese (*p* < 0.001), fresh fruit (*p* < 0.001), salted nuts (*p* = 0.008), unsalted nuts (*p* < 0.001), fresh vegetables (*p* < 0.001), beans and peas (*p* < 0.001), poultry (*p* = 0.004), fish (*p* < 0.001), shellfish (*p* < 0.001), and olive oil (*p* < 0.001) were more likely to have a moderate to high MD adherence. In addition, participants who consumed fresh fruit (*p* = 0.008), unsalted nuts (*p* = 0.001), fresh vegetables (*p* = 0.002), beans and peas (*p* < 0.001), poultry (*p* = 0.006), fish *(p* = 0.024), and olive oil (*p* = 0.001) more frequently were also found to be more likely to be moderately to highly MD-adherent in the multivariate logistic regression analysis. The multivariate logistic regression analysis also revealed that participants who drank wine more frequently were more likely to be moderately to strongly MD-adherent (Table 7).

### 3.5. The Influence of the MEDAS Score on the Anthropometric Parameters and Health Conditions of Subjects

In addition, a linear regression analysis was used to test the influence of the MEDAS score on the anthropometric parameters and health conditions of the subjects. Table 8 shows that the MEDAS score was associated with the body weight (*p* = 0.010). Participants with higher a MEDAS score had a lower body weight. On the other hand, the MEDAS score had no influence on the possibility of some health conditions.

## 4. Discussion

This cross-sectional study, conducted among the Slovenian population, tested the prevalence of adherence to the MD, and assessed the associations between sociodemographic and health variables and dietary habits with adherence to the MD. Women who had a higher education, lived in the western part of Slovenia, lived in a suburb or in the city, and were non-smokers were more likely to adhere to the MD. In addition, the results of the current work also show that a greater adherence to the MD is associated with the consumption of more fresh fruit and vegetables, beans, peas, poultry, fish, and olive oil and, on the other hand, with the consumption of less red meat, sausages, and butter. In general, the results show a low prevalence of moderate to good adherence to the MD in the Slovenian population. In light of this, the present results from the SI.Menu study represent the first attempt to identify the main determinants of the MD across Slovenia.

The benefits of the MD have been widely studied and documented [4,5,7,8,9,23] but, despite the proven health benefits, a recent decline in the adherence to the MD has been observed. Indeed, many studies have reported low to moderate MD adherence [14,15,24,25,26]. In our study, about half of the population rated their adherence to the MD as low (scores < 6). However, women had better adherence to the MD (55.6% moderately to strongly adhered to the MD) than men (42.6% moderately to strongly adhered to the MD). The results are consistent with another recent study of the Slovenian population [27]. In general, many studies report low prevalence rates of adherence to the MD [24,26]. The reasons for this decline are thought to be multifaceted. Current evidence suggests that adherence to the MD is slowly declining, mainly due to the increased availability of processed, ready-to-use, energy-dense foods, the globalised market, modern lifestyles, and a lack of time to prepare meals [28]. In addition, the global interest in dieting has increased, and people follow popular or trendy dietary patterns (such as the ketogenic diet, vegetarian diet, vegan diet, intermittent fasting, detox diet, etc.), assuming they will be a magic solution for their long-term problems, especially obesity [29]. It should be noted that these diets are normally very restrictive, and produce impressive results in a short time [30]. On the other hand, the MD is a moderate diet, and is not based on the complete restriction of a specific food group, but is instead characterized by a richness in plant-based food, and the moderation of refined grains, red meat, and dairy, and is therefore more effective in the long term [31]. Nevertheless, the rising cost of many essential foods to the MD has been suggested as a factor driving people to abandon this diet in favour of lower-cost, energy-dense foods, which tend to be of a lower nutritional quality [32]. Assessing adherence to the MD is not a common clinical practice, and there are no satisfying method to assess adherence to the MD. Indeed, in the last 25 years, an impressive number of different questionnaires have been proposed [10].

The MD is often characterized only in terms of food. It should be emphasized that the MD is more than just a diet. In addition to foods and meal patterns, there are other potential factors that could influence the implementation of the MD pattern [33]. Age, gender, socioeconomic status (education level, monthly income, and occupation), place of residence, healthy lifestyle behaviours (not smoking, higher physical activity) and marital status have also been studied [34]. Age and gender are factors that have been associated to varying degrees with adherence to the MD. Our results show that being female is one of the determinants of better adherence to the MD. Similar results have been found in other studies [23,35,36,37]. However, gender differences in the adherence to the MD have also been extensively studied, and the results are inconclusive. As mentioned above, some studies found a better adherence to the MD in women, some studies found no difference between men and women [5,38], and others found that women had a lower adherence than men [24,39,40,41]. In addition, the effect of age on adherence to the MD is still unclear. Some studies have reported a decrease in adherence with age, possibly due to a declining interest in eating, difficulty chewing, or financial difficulties after retirement [42]. Others found significantly lower scores in younger populations, as young people were more likely to accept foods from other cultures [41,43,44]. We need more insight into MD adherence scores across age ranges.

Higher education is generally associated with a healthier diet, and consumption of healthier foods [24]. Several studies have shown that higher levels of education are associated with better adherence to the MD [41,45,46]. Similar results were obtained in our study. A higher educational level was one of the predictors of a moderate to high adherence to the MD. However, in some studies, the educational level was not associated with the adherence to the MD [47,48].

Smoking is also one of the leading lifestyle risk factors [49]. It is well known that smokers usually have poorer nutrition than non-smokers [50]. Indeed, in our study, the smoking behaviour was one of the most important predictors of adherence to the MD. Being a non-smoker was associated with a better adherence to the MD. Our results are consistent with some previous studies showing that non-smokers or former smokers demonstrated higher adherence to the MD [14,37,46,48].

In our study, female gender, a high educational level, being a non-smoker, and living in a suburban or urban area and in the western part of Slovenia (with a narrow, indented coastline on the Adriatic Sea) were the most important factors for better adherence to the MD. Geographical location seems to be a very important factor contributing to compliance with the MD. Similar results were obtained by Ruggiero et al. [41], who found that Italians living in southern Italy were more compliant with the MD than northern Italians. Moreover, similar results were found in other studies [16,26]. In addition, we might expect the MD to be more prevalent in rural areas than in urban areas but, consistent with our results, Ruggiero et al. [41] also found that adherence to the MD was more prevalent in urban areas than in rural areas.

Other sociodemographic and lifestyle factors, such as physical activity, sleep quality, and marital status were not associated with adherence to the MD in our study. Our results are not consistent with other studies that have indicated that sleep duration, physical activity, and marital status are associated with adherence to the MD [51,52].

The impact of adherence to the MD on anthropometric parameters is also controversial. While some studies suggest that a higher adherence is associated with a lower BMI or body weight [53,54], others suggest the opposite [55,56]. In our study, an inverse relationship between adherence to the MD and body weight was found.

As mentioned earlier, the MD is more than just a diet. It represents social support, provides a sense of community, and can influence health status [57]. Indeed, the MD is widely recognized as a model of “healthy eating” because of its contribution to one’s health status, and its positive impact on quality of life [58]. In our study, better adherence to the MD was not significantly associated with the prevalence of hypertension, hypercholesterolemia, type 1 diabetes, type 2 diabetes, osteoporosis, colorectal cancer, and cardiovascular disease. However, similar to other studies, we only reported cross-sectional results, so further longitudinal studies appear to be necessary, to reassess the relationship between health status and adherence to the MD [59].

Regarding the frequency of consumption of different food groups, compared to men, women in our study consumed fresh fruit, vegetables, and olive oil more frequently, and pasta, pizza, red meat, convenience foods, hamburgers, sausages, and wine less frequently. The same was found by León-Muñoz. et al. [14]. The results may be explained by the fact that women tend to have healthier lifestyles, and are therefore more likely to adhere to the MD. Regarding wine consumption, only 5.6% of women drink wine 4–6 times per week or more, while 17.7% of men drink wine 4–6 times per week or more. The low wine consumption among women is related to the different habits of the genders. In general, the consumption of some foods included in the MD sample was lower than expected, and did not meet the national dietary recommendations [60]. The lowest compliance was found for the consumption of olive oil, fish, vegetables, and fruit. We also consume too much red meat and convenience foods, especially men (13% 4–6 times or more per week). The reasons why we do not adhere to the recommendation could be multiple. Firstly, in our study group, 60% of participants were retired, 7.4% were unemployed, and 4% were students. We are aware that some components of the MD are quite expensive, such as olive oil, fruit, and vegetables. In addition, almost 50% of our study participants had a low income. Previous studies have shown that low-income people are less likely to consume fruit and vegetables [17]. Therefore, further efforts should be considered by public health experts to promote healthy lifestyle habits in line with traditional dietary patterns. In addition, public policy should consider how to make some components of the MD, such as fruit and vegetables, more affordable for low-income families. The predictors of better adherence to the MD were: consumption of more fresh fruit, fresh vegetables, unsalted nuts, beans, peas, poultry, fish, and olive oil, and moderate wine consumption. On the other hand, the consumption of less butter, white bread, and convenience foods was also found to be associated with better adherence to the MD. The results were expected, as these food groups characterize the nutrition score used.

Several potential limitations and strengths of our study merit comment, and have been discussed elsewhere [61]. However, the main limitation is the cross-sectional nature of our analysis that does not allow the definition of causal relationships, but only associations. Secondly, the use of self-reported dietary data and questionnaires could result in underreporting or intentional measurement errors. Thirdly, because participation in the survey was voluntary, the participation rate was only 62%. Fourthly, we did not use household or other substitutions that would increase the participation rate. Fifthly, no financial compensation was given.

## 5. Conclusions

To the best of our knowledge, this study is the most recent investigation into the adherence to the MD at the national Slovenian level, and the assessment of associations between adherence to the MD and sociodemographic factors and dietary habits. We found that the prevalence of compliance with the MD in Slovenia is low. A better adherence to the MD is associated with female gender, a high education level, non-smoking status, living in a suburb or city, and living in the western part of Slovenia. In general, adherence to the MD in the Slovenian population is low. In addition, the consumption of some foods included in the MD sample was lower than expected, and did not meet the national dietary recommendations. Therefore, nutrition education campaigns should promote adherence to the MD, and raise awareness of the health benefits of good adherence to the recommendations, to support healthy ageing. In addition, our results should reach health centres and health promotion centres, to educate people, especially the less educated and those from rural areas, about the need to change their dietary habits, in accordance with the MD, for health, environmental, and climate reasons. Moreover, considering the fact that Slovenia is a Mediterranean country, the nutritional policy could aim to encourage people to eat more fruit and vegetables, and fish, especially in the eastern part of Slovenia, where adherence to the MD was shown to be lower than in the western part. Finally, facing the increasing rates of obesity and other diseases related to obesity, nowadays, many people follow trendy diets to improve their health and lose weight. In the short term, these diets are effective, and improve health to some extent. Nevertheless, long-term diets, such as the MD, that prescribe high-quality foods and have strong, evidence-based health and metabolic benefits, should be encouraged.

## Figures and Tables

**Table 1:** 14-point Mediterranean Diet Adherence Screener (MEDAS).

Questions	Criteria for 1 Point	Score
Do you use olive oil as the main culinary fat?	Yes	1
How much olive oil do you consume in a given day (including oil used for frying, salads, out-of-house meals, etc.)?	≥4 tbsp	1
How many vegetable servings do you consume per day? (1 serving: 200 g [consider side-dishes as half a serving])	≥2 (≥1 portion, raw or as a salad)	1
How many fruits (including natural fruit juices) do you consume per day? (One serving is 1/2 cup or a medium-sized piece of whole fruit)	≥3	1
How many servings of red meat, hamburger, or meat products (ham, sausage, etc) do you consume per day? (1 serving: 100–150 g)	<1	1
How many servings of butter, margarine, or cream do you consume per day (1 serving: 12 g)	<1	1
How many sweet or carbonated beverages do you drink per day?	<1	1
How much wine do you drink per week?	≥7 glasses	1
How many servings of legumes do you consume per week? (1 serving: 150 g)	≥3	1
How many servings of fish or shellfish do you consume per week? (1 serving: 100–150 g of fish or 4–5 units or 200 g of shellfish)	≥3	1
How many times per week do you consume commercial sweets or pastries (not homemade), such as cakes, cookies, biscuits, or custard?	<3	1
How many servings of nuts (including peanuts) do you consume per week? (1 serving: 30 g)	≥3	1
Do you preferentially consume chicken, turkey, or rabbit meat instead of veal, pork, hamburger, or sausage?	Yes	1
How many times per week do you consume vegetables, pasta, rice, or other dishes seasoned with sofrito (sauce made with tomato and onion, leek or garlic and simmered with olive oil)?	≥2	1
Total score		14

Note: tablespoon, tbsp.

**Table 2:** The demographic, anthropometric, socioeconomic, health condition, and lifestyle characteristics of the study sample, split by gender.

VARIABLES	All N (%) or M ± SD	Female N (%) or M ± SD	Male N (%) or M ± SD	*p* Value
	850	444 (52.2)	406 (47.8)	
**Age (years)**	57.0 ± 15.8	56.7 ± 17.1	57.4 ± 15.4	0.922
**BMI (kg/m^2^)**	27.8 ± 5.3	27.6 ± 5.9	27.9 ± 4.4	0.381
**Body weight (kg)**	79.3 ± 16.2	73.0 ± 15.4	86.1 ± 14.2	**<0.001**
**Body fat (%)**	31.6 ± 8.8	35.4 ± 7.9	27.4 ± 7.8	**<0.001**
**Physical activity**				0.870
>30 min/day	756 (88.9)	396 (89.2)	360 (88.7)	
<30 min/day	94 (11.1)	48 (10.8)	46 (11.3)	
**Family status**				**<0.001**
Single (single, widowed, divorced)	235 (27.7)	144 (32.4)	91 (22.4)	
In a relationship	614 (72.3)	300 (67.6)	314 (77.3)	
**Place of residence**				**0.007**
Village	466 (54.8)	220 (49.5)	246 (60.6)	
Suburban area	139 (16.4)	88 (19.8)	51 (12.6)	
City	245 (28.8)	136 (30.6)	109 (26.8)	
**Education**				0.568
No university degree	650 (76.5)	336 (75.7)	314 (77.3)	
University degree, Masters or Ph.D.	200 (23.5)	108 (24.3)	92 (22.7)	
**Geographical area**				0.122
East Slovenia	515 (60.6)	258 (58.1)	257 (63.3)	
West Slovenia	335 (39.4)	186 (41.9)	149 (36.7)	
**Smoking habits**				**<0.001**
Current	152 (17.9)	64 (14.4)	88 (21.7)	
Former	217 (25.5)	72 (16.2)	145 (35.7)	
No	481 (56.6)	308 (69.4)	197 (48.5)	
**Sleeping (h/day)**				0.255
During week	7.1 ± 1.4	7.2 ± 1.3	7.1 ± 1.5	
During weekend	7.5 ± 1.4	7.5 ± 1.3	7.4 ± 1.5	
**Employment status**				0.491
Employed	240 (28.3)	122 (27.5)	119 (29.3)	
Unemployed	64 (7.5)	39 (8.8)	25 (6.2)	
Student	34 (4.0)	21 (4.7)	13 (3.2)	
Retired	511 (60.2)	262 (59.0)	249 (61.5)	
**Family monthly net income (€)**				0.175
<1300	421 (49.5)	224 (50.4)	197 (48.5)	
1301–2500	265 (31.2)	148 (33.3)	138 (33.9)	
>2500	57 (6.7)	22 (4.9)	35 (8.6)	
Do not wish to tell or do not know	107 (12.6)	50 (11.4)	57 (14.0)	
**Health conditions**				
Hypertension				**0.040**
Yes	330 (38.8)	158 (35.6)	172 (42.4)	
No	517 (60.8)	285 (64.2)	232 (57.1)	
Hypercholesterolemia				0.145
Yes	228 (26.8)	128 (28.8)	100 (24.6)	
No	614 (72.2)	310 (69.8)	304 (74.9)	
Diabetes mellitus type 1				**0.001**
Yes	14 (1.6)	2 (0.5)	12 (3.0)	
No	827 (97.3)	436 (98.2)	391 (96.3)	
Diabetes mellitus type 2				0.387
Yes	64 (7.5)	30 (6.8)	34 (8.4)	
No	779 (91.6)	409 (92.1)	370 (91.1)	
Cardiovascular diseases				**0.033**
Yes	100 (11.8)	42 (9.5)	58 (14.3)	
No	742 (87.3)	396 (89.2)	346 (85.2)	
Osteoporosis				**<0.001**
Yes	54 (6.4)	49 (11.0)	5 (1.2)	
No	790 (92.9)	390 (87.8)	400 (98.5)	
Colon cancer				0.573
Yes	12 (1.4)	6 (1.4)	6 (1.5)	
No	833 (98.0)	434 (97.7)	399 (98.3)	
**MEDAS score (0–14)**	5.6 ± 2.1	5.9 ± 2.1	5.3 ± 2.1	**<0.001**
Adherence to the Mediterranean diet				**0.002**
Low (0–5)	430 (50.6)	197 (44.4)	233 (57.4)	
Moderate to high (6–14)	420 (49.4)	247 (55.6)	173 (42.6)	

Notes: Body mass index (BMI), mean (M), number (N), standard deviation (SD). Bold values indicate the statistical significance between women and men *p* ≤ 0.05.

**Table 3:** The frequency of consuming the most typical representatives from nine food groups, split by gender.

FOOD LIST	All N (%) or M ± SD	Female N (%) or M ± SD	Male N (%) or M ± SD	*p* Value
	850	444 (52.2)	406 (47.8)	
**CEREAL AND CEREAL PRODUCTS**				
**White bread**				**0.018**
*Never, 1–3 times per month or less*	301 (35.4)	175 (39.4)	126 (31.0)	
*Once per week, 2–3 times per week*	218 (25.6)	117 (26.4)	101 (24.9)	
*4–6 times per week*	86 (10.1)	37 (8.3)	49 (12.1)	
*1–2 times per day or more*	245 (28.8)	115 (25.9)	130 (32.0)	
**Brown bread**				0.927
*Never, 1–3 times per month or less*	176 (20.7)	89 (20.0)	87 (21.4)	
*Once per week, 2–3 times per week*	235 (27.6)	123 (27.7)	112 (27.6)	
*4–6 times per week*	127 (14.9)	65 (14.6)	62 (15.3)	
*1–2 times per day or more*	312 (36.7)	167 (37.6)	145 (35.7)	
**Rice**				0.696
*Never, 1–3 times per month or less*	227 (26.7)	124 (27.9)	103 (25.4)	
*Once per week, 2–3 times per week*	602 (70.8)	311 (70.0)	291 (71.7)	
*4–6 times per week*	17 (2.0)	7 (1.6)	10 (2.5)	
*1–2 times per day or more*	4 (0.5)	2 (0.5)	2 (0.5)	
**Pasta**				**0.031**
*Never, 1–3 times per month or less*	165 (19.4)	99 (22.3)	66 (16.3)	
*Once per week, 2–3 times per week*	662 (77.9)	338 (76.1)	324 (79.8)	
*4–6 times per week*	15 (1.8)	5 (1.1)	10 (2.5)	
*1–2 times per day or more*	8 (0.9)	2 (0.5)	6 (1.5)	
**MILK AND MILK PRODUCTS**				
**Milk**				**0.007**
*Never, 1–3 times per month or less*	217 (25.5)	104 (23.4)	113 (27.8)	
*Once per week, 2–3 times per week*	164 (19.3)	97 (17.8)	85 (20.9)	
*4–6 times per week*	83 (9.8)	35 (7.9)	48 (11.8)	
*1–2 times per day or more*	386 (45.4)	226 (50.9)	160 (39.4)	
**Yoghurt, soured milk, and cottage cheese**				**<0.001**
*Never, 1–3 times per month or less*	185 (21.8)	73 (16.4)	112 (27.6)	
*Once per week, 2–3 times per week*	393 (46.2)	212 (47.7)	181 (44.6)	
*4–6 times per week*	123 (14.5)	66 (14.9)	57 (14.0)	
*1–2 times per day or more*	149 (17.5)	93 (20.9)	56 (13.8)	
**Cheese**				**0.005**
*Never, 1–3 times per month or less*	190 (22.4)	117 (26.4)	73 (18.0)	
*Once per week, 2–3 times per week*	447 (52.6)	218 (49.1)	229 (56.4)	
*4–6 times per week*	135 (15.9)	62 (14.0)	73 (18.0)	
*1–2 times per day or more*	78 (9.2)	47 (10.6)	31 (7.6)	
**FRUITS**				
**Fresh fruits**				**<0.001**
*Never, 1–3 times per month or less*	29 (3.4)	11 (2.5)	18 (4.4)	
*Once per week, 2–3 times per week*	167 (19.6)	58 (3.1)	109 (26.8)	
*4–6 times per week*	148 (17.4)	74 (16.7)	74 (18.2)	
*1–2 times per day or more*	506 (59.5)	301 (67.8)	205 (50.5)	
**Salted nuts**				0.186
*Never, 1–3 times per month or less*	747 (87.9)	391 (88.1)	356 (87.7)	
*Once per week, 2–3 times per week*	89 (10.5)	42 (9.5)	47 (11.6)	
*4–6 times per week*	5 (0.6)	4 (0.9)	1 (0.2)	
*1–2 times per day or more*	9 (1.1)	7 (1.6)	2 (0.5)	
**Unsalted nuts**				**0.008**
*Never, 1–3 times per month or less*	633 (74.5)	311 (70.0)	322 (79.3)	
*Once per week, 2–3 times per week*	162 (19.1)	95 (21.4)	67 (16.5)	
*4–6 times per week*	23 (2.7)	17 (3.8)	6 (1.5)	
*1–2 times per day or more*	32 (3.8)	21 (4.7)	11 (2.7)	
**VEGETABLES and POTATOES**				
**Fresh vegetables**				**0.010**
*Never, 1–3 times per month or less*	47 (5.5)	18 (4.1)	29 (7.1)	
*Once per week, 2–3 times per week*	166 (19.5)	80 (18.0)	86 (21.2)	
*4–6 times per week*	206 (24.2)	98 (22.1)	108 (26.6)	
*1–2 times per day or more*	431 (50.7)	248 (55.9)	183 (45.1)	
**Legumes (beans, peas)**				0.346
*Never, 1–3 times per month or less*	173 (20.4)	89 (20.0)	84 (20.7)	
*Once per week, 2–3 times per week*	582 (68.5)	308 (69.4)	274 (67.5)	
*4–6 times per week*	70 (8.2)	31 (7.0)	39 (9.6)	
*1–2 times per day or more*	25 (2.9)	16 (3.6)	9 (2.2)	
**Potatoes**				0.348
*Never, 1–3 times per month or less*	44 (5.2)	28 (6.3)	16 (3.9)	
*Once per week, 2–3 times per week*	522 (61.4)	270 (60.8)	252 (62.1)	
*4–6 times per week*	239 (28.1)	120 (27.0)	119 (29.3)	
*1–2 times per day or more*	45 (5.3)	26 (5.9)	19 (4.7)	
**MEAT, FISH, AND MEAT PRODUCTS**				
**Poultry**				0.768
*Never, 1–3 times per month or less*	111 (13.1)	59 (13.3)	52 (12.8)	
*Once per week, 2–3 times per week*	658 (77.4)	346 (77.9)	312 (76.8)	
*4–6 times per week*	65 (7.6)	30 (6.8)	35 (8.6)	
*1–2 times per day or more*	16 (1.9)	9 (2.0)	7 (1.7)	
**Beef, veal**				**<0.001**
*Never, 1–3 times per month or less*	121 (14.2)	85 (19.1)	36 (8.9)	
*Once per week, 2–3 times per week*	651 (76.6)	335 (75.5)	316 (77.8)	
*4–6 times per week*	67 (7.9)	21 (4.7)	46 (11.3)	
*1–2 times per day or more*	11 (1.3)	3 (0.7)	8 (2.0)	
**Fish (sea)**				**0.044**
*Never, 1–3 times per month or less*	652 (76.7)	328 (73.9)	324 (79.8)	
*Once per week, 2–3 times per week*	195 (22.9)	113 (25.5)	82 (20.2)	
*4–6 times per week*	3 (0.4)	3 (0.7)	0 (0.0)	
*1–2 times per day or more*	0 (0.0)	0 (0.0)	0 (0.0)	
**Shellfish**				0.308
*Never, 1–3 times per month or less*	794 (93.4)	410 (92.3)	384 (94.6)	
*Once per week, 2–3 times per week*	55 (6.5)	33 (7.4)	22 (5.4)	
*4–6 times per week*	1 (0.1)	1 (0.2)	0 (0.0)	
*1–2 times per day or more*	0 (0.0)	0 (0.0)	0 (0.0)	
**Sausages, hot dogs**				**<0.001**
*Never, 1–3 times per month or less*	426 (50.1)	266 (59.9)	160 (39.4)	
*Once per week, 2–3 times per week*	384 (45.2)	166 (37.4)	218 (53.4)	
*4–6 times per week*	33 (3.9)	10 (2.3)	23 (5.7)	
*1–2 times per day or more*	7 (0.8)	2 (0.5)	5 (1.2)	
**Hamburger**				**<0.001**
*Never, 1–3 times per month or less*	805 (94.7)	434 (97.7)	371 (91.4)	
*Once per week, 2–3 times per week*	40 (4.7)	9 (2.0)	31 (7.6)	
*4–6 times per week*	3 (0.4)	0 (0.0)	3 (0.7)	
*1–2 times per day or more*	1 (0.1)	0 (0.0)	1 (0.2)	
**FATS AND FATTY FOOD**				
**Olive oil**				**0.004**
*Never, 1–3 times per month or less*	233 (27.4)	108 (24.3)	125 (30.8)	
*Once per week, 2–3 times per week*	196 (23.1)	94 (21.2)	102 (25.1)	
*4–6 times per week*	141 (16.6)	72 (16.2)	69 (17.0)	
*1–2 times per day or more*	279 (32.8)	170 (38.8)	109 (26.8)	
**Butter**				**0.002**
*Never, 1–3 times per month or less*	272 (32.0)	122 (27.5)	150 (36.9)	
*Once per week, 2–3 times per week*	405 (47.6)	213 (48.0)	192 (47.3)	
*4–6 times per week*	90 (10.6)	54 (12.2)	36 (8.9)	
*1–2 times per day or more*	83 (9.8)	55 (12.4)	28 (6.9)	
**Margarine**				0.753
*Never, 1–3 times per month or less*	633 (74.5)	326 (73.4)	307 (75.6)	
*Once per week, 2–3 times per week*	185 (21.8)	100 (22.5)	85 (20.9)	
*4–6 times per week*	17 (2.0)	9 (2.0)	8 (2.0)	
*1–2 times per day or more*	14 (1.6)	9 (2.0)	5 (1.2)	
**BEVERAGES**				
**Wine**				**<0.001**
*Never, 1–3 times per month or less*	518 (60.9)	319 (71.8)	199 (49.0)	
*Once per week, 2–3 times per week*	234 (27.5)	100 (22.5)	134 (33.0)	
*4–6 times per week*	44 (5.2)	13 (2.9)	31 (7.6)	
*1–2 times per day or more*	53 (6.2)	12 (2.7)	41 (10.1)	
**MISCELLANEOUS**				
**Prepared food**				**0.024**
*Never, 1–3 times per month or less*	775 (91.2)	414 (93.2)	361 (88.9)	
*Once per week, 2–3 times per week*	69 (8.1)	28 (6.3)	41 (10.1)	
*4–6 times per week*	4 (0.5)	0 (0.0)	4 (1.0)	
*1–2 times per day or more*	1 (0.1)	1 (0.2)	0 (0.0)	
**Burek, pizza**				0.168
*Never, 1–3 times per month or less*	712 (83.8)	380 (85.6)	332 (81.8)	
*Once per week, 2–3 times per week*	129 (15.2)	60 (13.5)	69 (17.0)	
*4–6 times per week*	5 (0.6)	1 (0.2)	4 (1.0)	
*1–2 times per day or more*	1 (0.1)	1 (0.2)	0 (0.0)	

**Notes**: Mean (M), number (N), standard deviation (SD). Bold values indicate the statistical significance between women and men *p* ≤ 0.05.

**Table 4:** The influence of the socio-demographic determinants of participants on their MEDAS score.

	ß	t	*p* Value
**VARIABLES**			
**Gender**	0.106	3.083	**0.002**
Age (years)	0.095	1.887	0.060
**Physical activity**	0.068	2.014	**0.044**
Sleeping (h)			
- Working days	0.032	1.110	0.390
- Per weekend	0.053	0.988	0.588
Family status	0.039	1.122	0.262
**Education**	0.128	3.649	**<0.001**
**Geographical area**	0.099	2.884	**0.004**
**Smoking habits**	−0.104	−2.986	**0.003**
Employment status	0.002	0.136	0.892
Family income	−0.053	−1.511	0.131

**Table 5:** The influence of the frequency of consumption of the different food groups on participants’ MEDAS scores.

	ß	t	*p* Value
**FOOD GROUPS**			
**White bread**	−0.061	−2.209	**0.027**
Brown bread	−0.012	−0.440	0.660
Rice	−0.010	−0.056	0.955
Pasta	−0.042	−1.689	0.092
Burek, pizza	−0.027	−1.083	0.279
Milk	−0.011	−0.513	0.608
Yoghurt, soured milk, cottage cheese	0.012	0.416	0.678
Cheese	0.039	1.638	0.102
**Fresh fruits**	0.229	9.546	**<0.001**
Salted nuts	0.042	1.750	0.081
**Unsalted nuts**	0.120	4.943	**0.006**
**Fresh vegetables**	0.140	5.940	**<0.001**
**Beans, peas**	0.200	8.838	**<0.001**
Potato	−0.034	−1.502	0.134
**Poultry**	0.094	4.053	**0.015**
**Beef, veal**	−0.086	−3.755	**<0.001**
**Sausages, hot dogs**	−0.080	−3.413	**<0.001**
**Fish (sea)**	0.067	2.761	**0.006**
Shellfish	0.006	0.250	0.803
Hamburgers	0.024	0.937	0.349
**Olive oil**	0.446	18.906	**<0.001**
**Butter**	−0.119	−5.176	**<0.001**
**Margarine**	−0.053	−2.382	**0.017**
**Wine**	0.115	5.083	**<0.001**
**Prepared food**	−0.045	−2.028	**0.043**

**Table 6:** Logistic regression analysis for having a moderate to high score of adherence to the Mediterranean diet, considering the assessed sociodemographic variables alone (univariate analysis) or together (multivariate analysis).

	Univariate Analysis		Multivariate Analysis	
	OR (95% Cl)	*p*	OR (95% Cl)	*p*
**FOOD CONSUMPTION**				
**Gender**				
Male	-1-		-1-	
Female	1.689 (1.287–2.216)	**<0.001**	1.534 (1.156–2.034)	**0.003**
**Physical activity**				
>30 min/day	-1-		-1-	
<30 min/day	0.698 (0.452–1.078)	0.105	0.818 (0.516–1.298)	0.353
**Place of residence**				
Village	-1-		-1-	
Suburban area	1.721 (1.174–2.522)	**0.005**	1.511 (1.016–2.249)	**0.042**
City	1.714 (1.254–2.342)	**<0.001**	1.568 (1.122–2.191)	**0.008**
**Education**				
No university degree	-1-		-1-	
University degree	1.703 (1.235–2.350)	**0.001**	1.527 (1.098–2.125)	**0.012**
**Geographical area**				
East Slovenia	-1-		-1-	
West Slovenia	1.690 (1.280–2.231)	**<0.001**	1.558 (1.170–2.074)	**0.002**
**Smoking habits**				
No	-1-		-1-	
Former	0.920 (0.668–1.269)	0.612	1.089 (0.767–1.547)	0.634
Current	0.471 (0.322–0.687)	**<0.001**	0.561 (0.380–0.830)	**0.004**

**Table 7:** Logistic regression analysis for having a moderate to high score of adherence to the MD, considering the frequency of consumption of the different food groups alone (univariate analysis) or together (multivariate analysis).

	Univariate Analysis		Multivariate Analysis	
	OR (95% Cl)	*p*	OR (95% Cl)	*p*
**VARIABLES**				
**CEREAL AND CEREAL PRODUCTS**				
**White bread**				
*Never, 1–3 times per month or less*	-1-		-1-	
*Once per week, 2–3 times per week*	0.722 (0.508–1.026)	0.070	0.811 (0.495–1.327)	0.404
*4–6 times per week*	0.429 (0.262–0.702)	**<0.001**	0.771 (0.390–1.523)	0.454
*1–2 times per day or more*	0.455 (0.322–0.642)	**<0.001**	0.810 (0.501–1.312)	0.392
**FRUITS**				
**Fresh fruits**				
*Never, 1–3 times per month or less*	-1-		-1-	
*Once per week, 2–3 times per week*	1.559 (0.598–4.069)	0.364	1.634 (0.419–6.373)	0.479
*4–6 times per week*	1.469 (0.558–3.867)	0.436	1.284 (0.327–5.039)	0.720
*1–2 times per day or more*	6.879 (2.750–17.205)	**<0.001**	6.631 (1.761–24.974)	**0.005**
**Unsalted nuts**				
*Never, 1–3 times per month or less*	-1-		-1-	
*Once per week, 2–3 times per week*	3.418 (2.348–4.974)	**<0.001**	2.870 (1.740–4.735)	**0.001**
**VEGETABLES**				
**Fresh vegetables**				
*Never, 1–3 times per month or less*	-1-		-1-	
*Once per week, 2–3 times per week*	1.304 (0.613–2.774)	0.491	1.405 (0.526–3.755)	0.498
*4–6 times per week*	1.796 (0.863–3.739)	0.117	1.627 (0.624–4.242)	0.319
*1–2 times per day or more*	6.662 (3.293–13.477)	**<0.001**	4.380 (1.719–11.162)	**0.002**
**Beans, peas**				
*Never, 1–3 times per month or less*	-1-		-1-	
*Once per week, 2–3 times per week*	2.211 (1.542–3.171)	**<0.001**	3.381 (1.994–5.735)	**<0.001**
*4–6 times per week*	7.933 (4.116–15.292)	**<0.001**	7.856 (1.964–31.431)	**<0.001**
**MEAT, FISH, AND MEAT PRODUCTS**				
**Poultry**				
*Never, 1–3 times per month or less*	-1-		-1-	
*Once per week, 2–3 times per week*	0.632 (0.421–0.947)	**0.026**	0.541 (0.294–0.996)	**0.048**
*4–6 times per week*	2.823 (1.400–5.690)	**0.004**	4.031 (1.490–10.908)	**0.006**
**Beef, veal**				
*Never, 1–3 times per month or less*	-1-		-1-	
*Once per week, 2–3 times per week*	0.327 (0.214–0.502)	**<0.001**	0.237 (0.127–0.441)	**<0.001**
*4–6 times per week*	0.467 (0.249–0.876)	**0.018**	0.343 (0.140–0.838)	**0.019**
**Fish**				
*Never, 1–3 times per month or less*	-1-			
*Once per week, 2–3 times per week*	2.672 (1.906–3.748)	**<0.001**	1.790 (1.080–2.967)	**0.024**
**Sausages, hot dogs**				
*Never, 1–3 times per month or less*	-1-		-1-	
*Once per week, 2–3 times per week*	0.429 (0.323–0.569)	**<0.001**	0.588 (0.391–0.884)	**0.011**
**FATS AND FATTY FOOD**				
**Olive oil**				
*Never, 1–3 times per month or less*	-1-		-1-	
*Once per week, 2–3 times per week*	3.666 (2.331–5.765)	**<0.001**	5.635 (3.199–9.926)	**<0.001**
*4–6 times per week*	5.084 (3.111–8.189)	**<0.001**	7.719 (4.172–14.279)	**<0.001**
*1–2 times per day or more*	26.695 (16.638–42.831)	**<0.001**	39.418 (20.933–74.224)	**<0.001**
**Butter**				
*Never, 1–3 times per month or less*	-1-		-1-	
*Once per week, 2–3 times per week*	0.906 (0.665–1.233)	0.528	0.461 (0.289–0.735)	**0.001**
*4–6 times per week*	0.793 (0.466–1.218)	0.248	0.367 (0.184–0.731)	**0.004**
*1–2 times per day or more*	1.001 (0.905–2.462)	0.116	0.332 (0.153–0.717)	**0.005**
**Margarine**				
*Never, 1–3 times per month or less*	-1-		-1-	
*Once per week, 2–3 times per week*	0.525 (0.375–0.735)	**<0.001**	0.645 (0.345–0.863)	0.884
**BEVERAGES**				
**Wine**				
*Never, 1–3 times per month or less*	-1-		-1-	
*Once per week, 2–3 times per week*	0.939 (0.689–1.280)	0.691	1.060 (0.676–1.661)	0.801
*4–6 times per week*	1.869 (0.988–3.539)	0.055	4.905 (2.119–11.356)	**<0.001**
*1–2 times per day or more*	1.505 (0.849–2.670)	0.162	2.755 (1.108–6.850)	0.055
**MISCELLANEOUS**				
**Prepared food**				
*Never, 1–3 times per month or less*	-1-		-1-	
*Once per week, 2–3 times per week*	0.478 (0.284–0.805)	**0.005**	0.647 (0.317–1.322)	0.232

**Table 8:** The influence of the MEDAS score on the body composition and health status of the participants.

	ß	t	*p* Value
**VARIABLES**			
**ANTHROPOMETRIC PARAMETERS**			
BMI (kg/m^2^)	−0.062	−1.807	0.071
Body weight (kg)	−0.089	−2.577	**0.010**
Body fat (%)	0.058	1.413	0.158
**HEALTH CONDITIONS**			
Hypertension	−0.042	−1.210	0.227
Hypercholesterolemia	0.030	0.879	0.380
Diabetes mellitus type 1	0.046	1.335	0.182
Diabetes mellitus type 2	−0.004	−0.108	0.914
Osteoporosis	0.024	0.692	0.489
Cardiovascular diseases	−0.003	−0.097	0.923
Colon cancer	0.013	0.380	0.704

## Data Availability

Data will be made available upon reasonable request to the corresponding authors.

## References

[1] Davis C., Bryan J., Hodgson J., Murphy K. (2015). Definition of the Mediterranean Diet; A Literature Review. Nutrients.

[2] Willett W.C., Sacks F., Trichopoulou A., Drescher G., Ferro-Luzzi A., Helsing E., Trichopoulos D. (1995). Mediterranean diet pyramid: A cultural model for healthy eating. Am. J. Clin. Nutr..

[3] Food and Agriculture Organization of the United Nations (FAO), World Health Organization (WHO) (2019). Sustainable Healthy Diets—Guiding Principles. Rome. https://www.who.int/publications/i/item/9789241516648.

[4] Liyanage T., Ninomiya T., Wang A., Neal B., Jun M., Wong M.G., Jardine M., Hillis G.S., Perkovic V. (2016). Effects of the Mediterranean Diet on Cardiovascular Outcomes—A Systematic Review and Meta-Analysis. PLoS ONE.

[5] Grosso G., Marventano S., Yang J., Micek A., Pajak A., Scalfi L., Galvano F., Kales S.N. (2017). A comprehensive meta-analysis on evidence of Mediterranean diet and cardiovascular disease: Are individual components equal?. Crit. Rev. Food Sci. Nutr..

[6] Dinu M., Pagliai G., Casini A., Sofi F. (2018). Mediterranean diet and multiple health outcomes: An umbrella review of meta-analyses of observational studies and randomised trials. Eur. J. Clin. Nutr..

[7] Eleftheriou D., Benetou V., Trichopoulou A., La Vecchia C., Bamia C. (2018). Mediterranean diet and its components in relation to all-cause mortality: Meta-analysis. Br. J. Nutr..

[8] Schwingshackl L., Schwedhelm C., Galbete C., Hoffmann G. (2017). Adherence to Mediterranean Diet and Risk of Cancer: An Updated Systematic Review and Meta-Analysis. Nutrients.

[9] Morze J., Danielewicz A., Przybyłowicz K., Zeng H., Hoffmann G., Schwingshackl L. (2021). An updated systematic review and meta-analysis on adherence to mediterranean diet and risk of cancer. Eur. J. Nutr..

[10] Chiriacò M., Tubili C., Bo S., Parillo M., Vetrani C., Mazzotti A., Pistis D., Marelli G., Grandone I., Natali A. (2023). Critical evaluation of the questionnaires assessing adherence to the Mediterranean diet that are based on servings. Nutr. Metab. Cardiovasc. Dis..

[11] Martínez-González M.A., García-Arellano A., Toledo E., Salas-Salvadó J., Buil-Cosiales P., Corella D., Covas M.I., Schröder H., Arós F., Gómez-Gracia E. (2012). A 14-Item Mediterranean Diet Assessment Tool and Obesity Indexes among High-Risk Subjects: The PREDIMED Trial. PLoS ONE.

[12] Schröder H., Fitó M., Estruch R., Martínez-González M.A., Corella D., Salas-Salvadó J., Lamuela-Raventós R., Ros E., Salaverría I., Fiol M. (2011). A short screener is valid for assessing Mediterranean diet adherence among older Spanish men and women. J. Nutr..

[13] da Silva R., Bach-Faig A., Raidó Quintana B., Buckland G., Vaz de Almeida M.D., Serra-Majem L. (2009). Worldwide variation of adherence to the Mediterranean diet, in 1961–1965 and 2000–2003. Public Health Nutr..

[14] León-Muñoz L.M., Guallar-Castillón P., Graciani A., López-García E., Mesas A.E., Aguilera M.T., Banegas J.R., Rodríguez-Artalejo F. (2012). Adherence to the Mediterranean diet pattern has declined in Spanish adults. J. Nutr..

[15] Vilarnau C., Stracker D.M., Funtikov A., da Silva R., Estruch R., Bach-Faig A. (2019). Worldwide adherence to Mediterranean Diet between 1960 and 2011. Eur. J. Clin. Nutr..

[16] Mattavelli E., Olmastroni E., Bonofiglio D., Catapano A.L., Baragetti A., Magni P. (2022). Adherence to the Mediterranean Diet: Impact of Geographical Location of the Observations. Nutrients.

[17] Oude Groeniger J., van Lenthe F.J., Beenackers M.A., Kamphuis C.B. (2017). Does social distinction contribute to socioeconomic inequalities in diet: The case of ‘superfoods’ consumption. Int. J. Behav. Nutr. Phys. Act..

[18] Rush E., Yan M. (2017). Evolution not Revolution: Nutrition and Obesity. Nutrients.

[19] World Health Organization State of Health in the EU Slovenia Country Health Profile 2019. https://eurohealthobservatory.who.int/publications/m/slovenia-country-health-profile-2019.

[20] European Food Safety Authority (2014). Guidance on the EU Menu methodology. EFSA J..

[21] Gregorič M., Blaznik U., Delfar N., Zaletel M., Lavtar D., Koroušić Seljak B., Golja P., Zdešar Kotnik K., Pravst I., Fidler Mis N. (2019). Slovenian national food consumption survey in adolescents, adults and elderly. EFSA Support. Publ..

[22] García-Conesa M.-T., Philippou E., Pafilas C., Massaro M., Quarta S., Andrade V., Jorge R., Chervenkov M., Ivanova T., Dimitrova D. (2020). Exploring the Validity of the 14-Item Mediterranean Diet Adherence Screener (MEDAS): A Cross-National Study in Seven European Countries around the Mediterranean Region. Nutrients.

[23] Dinu M., Pagliai G., Angelino D., Rosi A., Dall’Asta M., Bresciani L., Ferraris C., Guglielmetti M., Godos J., Del Bo’ C. (2020). Effects of Popular Diets on Anthropometric and Cardiometabolic Parameters: An Umbrella Review of Meta-Analyses of Randomized Controlled Trials. Adv. Nutr..

[24] Downer M.K., Gea A., Stampfer M., Sánchez-Tainta A., Corella D., Salas-Salvadó J., Ros E., Estruch R., Fitó M., Gómez-Gracia E. (2016). Predictors of short- and long-term adherence with a Mediterranean-type diet intervention: The PREDIMED randomized trial. Int. J. Behav. Nutr. Phys. Act..

[25] Ruggiero E., Mignogna C., Costanzo S., Persichillo M., Di Castelnuovo A., Esposito S., Cerletti C., Donati M.B., de Gaetano G., Iacoviello L. (2021). Changes in the consumption of foods characterising the Mediterranean dietary pattern and major correlates during the COVID-19 confinement in Italy: Results from two cohort studies. Int. J. Food Sci. Nutr..

[26] Obeid C.A., Gubbels J.S., Jaalouk D., Kremers S.P.J., Oenema A. (2022). Adherence to the Mediterranean diet among adults in Mediterranean countries: A systematic literature review. Eur. J. Nutr..

[27] Molina-Montes E., Uzhova I., Verardo V., Artacho R., García-Villanova B., Jesús Guerra-Hernández E., Kapsokefalou M., Malisova O., Vlassopoulos A., Katidi A. (2021). Impact of COVID-19 confinement on eating behaviours across 16 European countries: The COVIDiet cross-national study. Food Qual. Prefer..

[28] Srour B., Fezeu L.K., Kesse-Guyot E., Allès B., Debras C., Druesne-Pecollo N., Chazelas E., Deschasaux M., Hercberg S., Galan P. (2020). Ultraprocessed Food Consumption and Risk of Type 2 Diabetes Among Participants of the NutriNet-Santé Prospective Cohort. JAMA Intern. Med..

[29] Tahreem A., Rakha A., Rabail R., Nazir A., Socol C.T., Maerescu C.M., Aadil R.M. (2022). Fad Diets: Facts and Fiction. Front. Nutr..

[30] Ge L., Sadeghirad B., Ball G.D.C., Da Costa B.R., Hitchcock C.L., Svendrovski A., Kiflen R., Quadri K., Kwon H.Y., Karamouzian M. (2020). Comparison of dietary macronutrient patterns of 14 popular named dietary programmes for weight and cardiovascular risk factor reduction in adults: Systematic review and network meta-analysis of randomised trials. BMJ.

[31] Freire R. (2020). Scientific evidence of diets for weight loss: Different macronutrient composition, intermittent fasting, and popular diets. Nutrition.

[32] Lopez C.N., Martinez-Gonzalez M.A., Sanchez-Villegas A., Alonso A., Pimenta A.M., Bes-Rastrollo M. (2009). Costs of Mediterranean and western dietary patterns in a Spanish cohort and their relationship with prospective weight change. J. Epidemiol. Community Health.

[33] Tessier S., Gerber M. (2005). Comparison between Sardinia and Malta: The Mediterranean diet revisited. Appetite.

[34] Tsofliou F., Vlachos D., Hughes C., Appleton K.M. (2022). Barriers and Facilitators Associated with the Adoption of and Adherence to a Mediterranean Style Diet in Adults: A Systematic Review of Published Observational and Qualitative Studies. Nutrients.

[35] Aggarwal A., Monsivais P., Cook A.J., Drewnowski A. (2011). Does diet cost mediate the relation between socioeconomic position and diet quality?. Eur. J. Clin. Nutr..

[36] Pavičić Žeželj S., Kenđel Jovanović G., Dragaš Zubalj N., Mićović V., Sesar Ž. (2018). Associations between Adherence to the Mediterranean Diet and Lifestyle Assessed with the MEDLIFE Index among the Working Population. Int. J. Environ. Res. Public Health..

[37] Cuschieri S., Libra M. (2020). Adherence to the Mediterranean Diet in Maltese Adults. Int. J. Environ. Res. Public Health.

[38] Abellán Alemán J., Zafrilla Rentero M., Montoro-García S., Mulero J., Pérez Garrido A., Leal M., Guerrero L., Ramos E., Ruilope L. (2016). Adherence to the “Mediterranean Diet” in Spain and Its Relationship with Cardiovascular Risk (DIMERICA Study). Nutrients.

[39] Zazpe I., Estruch R., Toledo E., Sánchez-Taínta A., Corella D., Bulló M., Fiol M., Iglesias P., Gómez-Gracia E., Arós F. (2010). Predictors of adherence to a Mediterranean-type diet in the PREDIMED trial. Eur. J. Nutr..

[40] Bautista-Castaño I., Molina-Cabrillana J., Montoya-Alonso J.A., Serra-Majem L. (2004). Variables predictive of adherence to diet and physical activity recommendations in the treatment of obesity and overweight, in a group of Spanish subjects. Int. J. Obes..

[41] Ruggiero E., Di Castelnuovo A., Costanzo S., Persichillo M., Bracone F., Cerletti C., Donati M.B., de Gaetano G., Iacoviello L., Bonaccio M. (2019). Socioeconomic and psychosocial determinants of adherence to the Mediterranean diet in a general adult Italian population. Eur. J. Public Health.

[42] Foscolou A., D’Cunha N.M., Naumovski N., Tyrovolas S., Chrysohoou C., Rallidis L., Polychronopoulos E., Matalas A.L., Sidossis L.S., Panagiotakos D. (2020). The association between the level of adherence to the Mediterranean diet and successful aging: An analysis of the ATTICA and MEDIS (MEDiterranean Islands Study) epidemiological studies. Arch. Gerontol. Geriatr..

[43] Bonaccio M., Bes-Rastrollo M., de Gaetano G., Iacoviello L. (2016). Challenges to the Mediterranean diet at a time of economic crisis. Nutr. Metab. Cardiovasc. Dis..

[44] Veronese N., Notarnicola M., Cisternino A.M., Inguaggiato R., Guerra V., Reddavide R., Donghia R., Rotolo O., Zinzi I., Leandro G. (2020). Trends in adherence to the Mediterranean diet in South Italy: A cross sectional study. Nutr. Metab. Cardiovasc. Dis..

[45] Papadaki A., Wood L., Sebire S.J., Jago R. (2015). Adherence to the Mediterranean diet among employees in South West England: Formative research to inform a web-based, work-place nutrition intervention. Prev. Med. Rep..

[46] Marventano S., Godos J., Platania A., Galvano F., Mistretta A., Grosso G. (2018). Mediterranean diet adherence in the Mediterranean healthy eating, aging and lifestyle (MEAL) study cohort. Int. J. Food. Sci. Nutr..

[47] Tur J.A., Romaguera D., Pons A. (2004). Adherence to the Mediterranean dietary pattern among the population of the Balearic Islands. Br. J. Nutr..

[48] Navarro-Martínez R., Mafla-España M.A., Cauli O. (2022). Mediterranean Diet Adherence in Community-Dwelling Older Adults in Spain: Social Determinants Related to the Family. Nutrients.

[49] World Healrh Organization (2022). Tobacco. https://www.who.int/news-room/fact-sheets/detail/tobacco.

[50] Pardavila-Belio M.I., de la O V., Hershey M.S., Barbería-Latasa M., Toledo E., Martin-Moreno J.M., Martínez-González M., Ruiz-Canela M. (2022). Joint association of the Mediterranean diet and smoking with all-cause mortality in the Seguimiento Universidad de Navarra (SUN) cohort. Nutrition.

[51] Campanini M.Z., Guallar-Castillón P., Rodríguez-Artalejo F., Lopez-Garcia E. (2017). Mediterranean Diet and Changes in Sleep Duration and Indicators of Sleep Quality in Older Adults. Sleep.

[52] Godos J., Grosso G., Ferri R., Caraci F., Lanza G., Al-Qahtani W.H., Caruso G., Castellano S. (2023). Mediterranean diet, mental health, cognitive status, quality of life, and successful aging in southern Italian older adults. Exp. Gerontol..

[53] Mistretta A., Marventano S., Platania A., Godos J., Galvano F., Grosso G. (2017). Metabolic profile of the Mediterranean healthy Eating, Lifestyle and Aging (MEAL) study cohort. Mediterr. J. Nutr. Metab..

[54] Bertoli S., Leone A., Vignati L., Bedogni G., Martínez-González M., Bes-Rastrollo M., Spadafranca A., Vanzulli A., Battezzati A. (2015). Adherence to the Mediterranean diet is inversely associated with visceral abdominal tissue in Caucasian subjects. Clin. Nutr..

[55] Sánchez-Villegas A., Bes-Rastrollo M., Martínez-González M.A., Serra-Majem L. (2006). Adherence to a Mediterranean dietary pattern and weight gain in a follow-up study: The SUN cohort. Int. J. Obes..

[56] Li Y., Roswall N., Ström P., Sandin S., Adami H.-O., Weiderpass E. (2015). Mediterranean and Nordic diet scores and long-term changes in body weight and waist circumference: Results from a large cohort study. Br. J. Nutr..

[57] Bach-Faig A., Berry E.M., Lairon D., Reguant J., Trichopoulou A., Dernini S., Medina F.X., Battino M., Belahsen R., Miranda G. (2011). Mediterranean diet pyramid today. Science and cultural updates. Public Health Nutr..

[58] Tuttolomondo A., Simonetta I., Daidone M., Mogavero A., Ortello A., Pinto A. (2019). Metabolic and Vascular Effect of the Mediterranean Diet. Int. J. Mol. Sci..

[59] Álvarez-Álvarez I., Martínez-González M., Sánchez-Tainta A., Corella D., Díaz-López A., Fitó M., Vioque J., Romaguera D., Martínez J.A., Wärnberg J. (2019). Adherence to an Energy-restricted Mediterranean Diet Score and Prevalence of Cardiovascular Risk Factors in the PREDIMED-Plus: A Cross-sectional Study. Rev. Esp. Cardiol..

[60] NIJZ Referenčne Vrednosti za Energijski vnos ter vnos Hranil. Tabelarična Priporočila za Otroke (od 1. leta Starosti Naprej), Mladostnike, Odrasle, Starejše Odrasle, Nosečnice ter Doječe Matere. Dopolnjena Izdaja 2020. https://nijz.si/wp-content/uploads/2020/04/referencne_vrednosti_2020_3_2.cleaned.pdf.

[61] Seljak B.K., Valenčič E., Hristov H., Hribar M., Lavriša Ž., Kušar A., Žmitek K., Krušič S., Gregorič M., Blaznik U. (2021). Inadequate Intake of Dietary Fibre in Adolescents, Adults, and Elderlies: Results of Slovenian Representative SI. Menu Study. Nutrients.

